# Co thin films deposited directly on ZnO polar surfaces

**DOI:** 10.1038/srep38005

**Published:** 2016-11-29

**Authors:** Daichi Chiba, Naoya Shibata, Atsushi Tsukazaki

**Affiliations:** 1 Department of Applied Physics, The University of Tokyo, Bunkyo, Tokyo 113-8656, Japan; 2 Institute of Engineering Innovation, The University of Tokyo, Bunkyo, Tokyo 113-8656, Japan; 3 Institute for Materials Research, Tohoku University, Sendai 980-8577, Japan; 4 PRESTO, Japan Science and Technology Agency (JST), Chiyoda, Tokyo 102-0075, Japan

## Abstract

A ferromagnetic (FM)-metal/oxide stack is the key structure determining the performance of spintronic devices. However, the effect of the electronic polarity of the oxide on the magnetic properties of the adjacent FM-metal has not been investigated previously. Here, we report the magnetic and structural properties of Co ultra-thin films sputter deposited directly on the Zn- and O-polar surfaces of ZnO substrates. The magnetic anisotropy and Curie temperature exhibit dramatic polarity-dependent differences for films on these surfaces. Structural analyses reveal that the heterointerface of the Co/O-polar surface is rather diffusive, whereas that of the Co/Zn-polar surface is atomically flat. These results suggest that the surface polarity plays a key role in determining the properties of the film. This novel FM-metal/polar-oxide system is expected to add new functionality to spintronic devices and provide an ideal basis for investigating the effect of a built-in electric field on the magnetism in a metallic monolayer.

The combination of a ferromagnetic (FM) thin metal with a non-magnetic (NM) oxide layer has played a central role in the recent development of spintronic devices. For example, a single-crystal MgO layer interfaced with a 3d-FM metal results in giant tunnel magnetoresistance^1^,^2^ and perpendicular magnetic anisotropy (PMA) in the FM metal layer^3^. The ferromagnet/oxide heterostructure is also crucial for studies of the effects of an electric field on magnetism^4^,^5^,^6^,^7^,^8^,^9^,^10^ and novel phenomena or the magnetic structure in systems with structure inversion asymmetry^11^,^12^,^13^,^14^,^15^. Although the spin-filtering effect in tunnel junctions with an oxide multiferroic barrier has been reported^16^, the magnetism of a FM metal deposited directly on a polar oxide with a normal dielectric property has not been intensively explored. Thus, this system may present an exciting opportunity to add further magnetic functionality or controllability to FM metals.

To date, the polarity of a material has been positively used in oxide-oxide combinations. The polar discontinuity at perovskite interfaces can be a source of nontrivial local atomic and electronic structure^17^,^18^,^19^. In addition to perovskite interfaces, ZnO/MgZnO polar interfaces yield high-mobility two-dimensional electron gas without impurity doping or the application of external electric fields^20^,^21^. Here, we focus on the combination of a FM metal and a ZnO substrate with Zn- and O-polar surfaces (Fig. 1a) at its front and back sides, respectively, because of the broken inversion symmetry in the wurtzite crystal structure along the [0001] direction^22^. The surface termination of ZnO substrates also depends on the (0001) or (000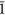) surface plane corresponding to the Zn-polar and O-polar faces, respectively^23^. When a FM metal is deposited directly on its surfaces, a built-in electric field should be introduced at the interface FM atoms, whose directions depend on the polar surface termination, as schematically shown in Fig. 1b. In this system, the built-in electric field effect on the magnetic properties (*e.g*., polarity-dependent magnetic anisotropy^7^,^24^, Curie temperature *T*_C_^8^,^25^, or Rashba spin-orbit-related phenomena^11^,^13^) is expected. In addition to its fundamental significance, exploring and developing such a system would provide new insights into material design in future spintronic devices. In this study, we investigate the magnetic and structural properties of FM Co thin films deposited directly on different ZnO polar surfaces. Dramatically different magnetic properties were observed for ZnO surfaces with different polarities.

Sets of samples with three different nominal Co layer thicknesses (*t*_Co_ = 0.40, 0.48, and 0.60 nm) were deposited on Zn- and O-polar surfaces (see Methods). For the structural analyses, a sample with a thickness *t*_Co_ of 3.6 nm was also prepared. All samples were capped by a 2.4-nm-thick Pt layer. The magnetic moment *m* for each sample was measured using a superconducting quantum interference device (SQUID) magnetometer (Magnetic Property Measurement System, Quantum Design inc.). The anomalous Hall effect, where the Hall resistance *R*_Hall_ is proportional to the perpendicular component of the magnetization, was also used to determine the magnetic properties of each sample (see Methods). The surface and structural characteristics of the samples were determined by x-ray diffraction (XRD) (Empyrean, PANalytical), atomic force microscopy (AFM) (AFM5000, Hitachi High-Technologies Cooporation), and scanning transmission electron microscopy (STEM). The possibility of intermixing between the layers and the substrate was examined by STEM-energy-dispersive x-ray spectroscopy (STEM-EDS). The scanning transmission electron microscope used in this study was aberration-corrected JEM-ARM200F (200 keV) equipped with Centurio SDD EDS detector (JEOL Co. Ltd.).

The magnetic properties differed dramatically depending on the polarity of the ZnO surface. Figure 2a and b show the *m*/*S*-*μ*_0_*H* curves for Zn- and O- polar samples, respectively, with *t*_Co_ = 0.4 nm, where *μ*_0_*H* and *S* are an external magnetic field and the area of the sample piece, respectively. Both the in-plane and perpendicular directions of *m* were measured to determine the magnetic easy axis of the sample. The magnetic anisotropy differs clearly between the samples types; *i.e*., the Zn-polar sample has an in-plane magnetic easy axis, whereas the O-polar sample clearly shows PMA. The dependence of the saturation *m* per area (*m*_s_/*S*) on *t*_Co_ at 10 K for the three samples with *t*_Co_ = 0.4–0.6 nm is plotted in Fig. 2c. The *m*_s_/*S* of both types of samples increases linearly with increasing *t*_Co_. The dashed-line in Fig. 2c is the linear fit to the data. The intercept of the linear fit was found to be non-zero, indicating an induced magnetic moment in the Pt cap layer attributed to the FM proximity effect^26^.

In addition to magnetic anisotropy, *T*_C_ also differed between the two types of samples. Figure 2d and e show the temperature dependence of the remanent magnetic moment *m*_r_ for both types of samples. A sharp transition from the FM state to the paramagnetic state is observed in the *m*_r_-*T* curves measured along the easy axis. No spin-reorientation transition (switching of the easy axis) is observed in either sample at any temperature. Figure 2f summarizes the *T*_C_ for Zn- and O-polar samples, where *T*_C_ is defined as the temperature at which *m*_r_ became zero along the easy axis. Based on this result, the *T*_C_ of the Zn-polar sample is higher (by approximately 60 K) than that of the O-polar sample. Notably, the two-dimensionality effect explains the increase of *T*_C_ with *t*_Co_, and its value is much lower than that in bulk-Co (~1400 K)^27^.

The value of *R*_Hall_ was measured to determine the magnetic anisotropy energy of the samples. Figure 3a shows the normalized *R*_Hall_ (*R*_Hall_^n^)-*μ*_0_*H*_⊥_ curve for three O-polar samples with *t*_Co_ = 0.4–0.6 nm at 10 K, where *H*_⊥_ is the magnetic field applied perpendicular to the film plane. The coercivity decreases with increasing *t*_Co_, suggesting that the PMA energy decreases with increasing *t*_Co_. The inset shows the *R*_Hall_^n^ curve for the sample with *t*_Co_ = 0.6 nm obtained as the magnetic field increased along the hard-axis (in-plane) direction (*H*_||_). *R*_Hall_^n^ decreased with *μ*_0_*H*_||_, showing that the magnetization of the sample tilts from being perpendicular to the plane to the in-plane direction. Based on the *R*_Hall_^n^-*μ*_0_*H*_||_ curve, the normalized magnetization along the hard axis (*M*_hard_^n^) is obtained using the following relationship: *M*_hard_^n^ = sin[arccos(*R*_Hall_^n^)](refs 28, 29, 30). The results at 10 K for the three samples are shown in Fig. 3b. The saturation field of *M*_hard_^n^ decreases with increasing *t*_Co_, in agreement with the coercivity variation with respect to *t*_Co_. The data shown in Fig. 3c of *M*_hard_^n^ for Zn-polar samples at 10 K presents the same trend for the saturation field. Here, *M*_hard_^n^ is the same as *R*_Hall_^n^ because the perpendicular direction is the hard axis for the Zn-polar samples.

The magnetic anisotropy energy per unit area *E*_⊥_*t*_Co_, where *E*_⊥_ is the effective perpendicular anisotropy energy density, is determined from the hard axis magnetization curves using the following equation (see for example ref. 28):


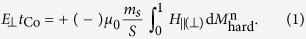


The values of *E*_⊥_*t*_Co_ as a function of *t*_Co_ for the Zn- and O-polar samples at 10 K are plotted in Fig. 3d, showing that the *E*_⊥_*t*_Co_ is linearly proportional to *t*_Co_. *E*_⊥_*t*_Co_ of the present system can be expressed as follows (see for example ref. 7).


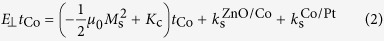


where *M*_s_ and *K*_c_ are the saturation magnetization and the uniaxial crystalline anisotropy energy constant, respectively. *k*_s_^ZnO/Co^ and *k*_s_^Co/Pt^ are the surface magnetic anisotropies attributed to the ZnO/Co and Co/Pt interfaces, respectively; their sum can be obtained from the intercept of the linear fitting in Fig. 3d. The slope of the linear fitting gives the sum of the demagnetization energy [(−1/2)*μ*_0_*M*_s_^2^] and *K*_c_. Based on these results, *k*_s_^ZnO/Co^ + *k*_s_^Co/Pt^ = 1.1 mJ/m^2^ and (−1/2)*μ*_0_*M*_s_^2^ + *K*_c_ = −1.2 MJ/m^3^ are obtained in the O-polar sample. The surface and crystalline anisotropies are approximately two or more times larger than the previously reported values determined in a Co/Pt system at room temperature^31^,^32^. However, the signs of the energy sums in the Zn-polar sample are opposite (−1.2 mJ/m^2^ and 0.48 MJ/m^3^, respectively). This result indicates that if *k*_s_^Co/Pt^ is positive (as in the general case), then *k*_surf._^ZnO/Co^ will be negative in the Zn-polar sample. In addition, *K*_c_ (Zn-polar) > (1/2)*μ*_0_*M*_s_^2^ > *K*_c_ (O-polar).

Importantly, the Co thin films deposited on the same material (ZnO) but with different polarization show completely different magnetic properties. To understand this phenomenon, the structural properties were investigated. Figure 4a shows the XRD profiles for Zn- and O-polar samples with *t*_Co_ = 3.6 and 0.6 nm obtained by the *θ*-2*θ* method using Cu-*Kα*_1_ radiation (wavelength: 0.15406 nm). The vertical dashed and dotted lines indicate the expected positions of the diffraction peak for the (111) plane of fcc Pt and Co crystals, respectively. Notably, the peak expected from Co with hcp (0002) structure resides at almost the same position as that of fcc(111). The Zn-polar sample with thicker *t*_Co_ (=3.6 nm) has clear peaks from the Pt and Co layers close to the expected positions. In addition, the thickness fringe is observed at the lower 2*θ* range (<10^o^), suggesting that sharp interfaces are formed. In contrast, no thickness fringe and no clear peaks other than the peak from the ZnO substrate are observed in the O-polar sample with *t*_Co_ = 3.6 nm. In samples with smaller *t*_Co_ values (=0.6 nm), the clear peak expected from Pt(111) and the thickness fringe are observed in both types of samples. A peak near the position expected from Co(111) is observed only in the Zn-polar sample, suggesting that the intermixing between Co and Pt is minor in this sample.

The AFM images for both samples are shown in Fig. 4b. In the Zn-polar sample with thicker *t*_Co_ (=3.6 nm), a flat surface with an average roughness height (*R*_a_) of 0.10 nm is observed. The O-polar sample shows a rough surface with *R*_a_ = 0.20 nm, in agreement with the XRD result. The *R*_a_ obtained in samples with thinner *t*_Co_ (=0.4 nm) is lower than one-monolayer (ML)-thick Co, showing that the sample surfaces are atomically flat. In the Zn-polar sample, an atomic step and terrace remain, even after the deposition of the Co/Pt layers.

The STEM images of the Zn- and O- polar samples with *t*_Co_ = 3.6 and 0.6 nm are summarized in Fig. 5. Samples with thicker *t*_Co_ (=3.6 nm) are discussed first (Fig. 5a and b). The Co and Pt layers on the Zn-polar surface have abrupt interfaces and much higher crystallinity than those on the O-polar sample, as expected from the XRD profiles. Figure 6a shows the magnified STEM image for the Zn-polar sample. The averaged results of the two-dimensional fast Fourier transformation (2D FFT) for the square areas indicated by the coloured squares in Fig. 6a are shown in Fig. 6b–d. Judging from the experimental (Fig. 6b–d) and simulated 2D FFT (Fig. 6e and f), the Pt layer (blue square) and the Co layer near the Co/Pt interface (light-green square) have a fcc(111) structure. Alternatively, a hcp(0001) texture appears to be dominant in the Co layer near the ZnO/Co interface (pink square) excluding the first ML just above the Zn-polar surface (we call the corresponding atomic layer “interface Co”). The interface Co atoms shows no dislocations in the observed length scale (over ~16 nm). Moreover, the number of atoms in the interface Co is exactly the same as that of the surface Zn atoms, indicating that the lateral atomic distances (LADs) of the Co and Zn atoms are almost equal (~0.32 nm). Therefore, in the Zn-polar sample, the LAD of the interface Co is approximately 30% larger than the bulk value. As shown in Fig. 7b, the in-plane LAD of each atomic layer was calculated by performing the FFT for each lateral pixel of the corresponding STEM image (Fig. 7a). The result evidences the fact that the LADs of the interface Co and Zn atoms are the same. From Fig. 7b and c(averaged intensity of the lateral pixels of STEM image in Fig. 7a), the lattice relaxation in the Co layer occurs in the first 2–3 MLs. The reasons that we can decide the ZnO/Co interface from the STEM image are the follows: (i) A clear dark region (gap) is observed just below the corresponding atomic layer in the STEM image (Fig. 7c). (ii) The atomic images below the “interface Co” has an ellipse-like shape, suggesting the existence of tilted Zn-O bonding, whereas the those of the “interface Co” atoms shows a spherical shape. Figure. 7d and e depict the schematic image of the position of the Co atoms on the Zn-polar surface expected based on the STEM image. The Co atom prefers the H_3_ site over the T_4_ or on-top of the Zn atoms. This observation is partially consistent with the result of an ab initio calculation^33^, in which the Co atom adsorbed on a Zn-polar surface avoids the on-top position.

The Co layer on the O-polar surface (Fig. 5b) shows an amorphous or poly-crystalline texture with rough interfaces with both the ZnO surface and the Pt layer. In both samples with thinner *t*_Co_ (=0.6 nm) (Fig. 5c and d), a clear fcc(111) texture is observed in the Pt layer. The darker region on the ZnO surface likely indicates Co atoms. The Pt/Co interface is not sharp, even in the Zn-polar sample, probably because the Co on the surface exhibited a non-perfect fcc structure at this thickness, as mentioned above.

The ZnO/Co interface in the O-polar samples (Fig. 5b and d) does not appear to be sharper than that in the Zn-polar samples (Fig. 5a and c). This phenomenon may be attributed to the mutual diffusion of Co and ZnO. To determine the atom distribution, STEM-EDS was performed. Figure 8a and b show the results of the STEM-EDS mapping of each element in the Zn- and O-polar samples with *t*_Co_ = 3.6 nm, respectively. The graphs on the right show the averaged line profiles along the deposition direction for each detected element in atomic %. In addition, a sharp ZnO/Co interface is observed in the Zn-polar sample. In the O-polar sample, clear intermixing between Co and O atoms occurs, particularly in the vicinity of the ZnO/Co interface. One possibility is that the mutual diffusion of Co and O facilitates the formation of Co-O bonds^34^ or decreases the surface-formation energy of the absorbed Co on the O-terminated surface^35^. The much weaker tolerance of the O-polar surface for chemical etching^36^ may be related to this phenomenon. Notably, the thermal stability of the Pd/Zn polar interface was experimentally confirmed using the polar interfaces of ZnO precipitates distributed in a single-crystal Pd matrix^37^,^38^. Nevertheless, further studies, including investigations of the nontrivial effects attributed to the polarity of the ZnO surface, such as polar discontinuity^18^, may be required to clarify the structural difference.

Finally, we comprehensively discuss the differences in the observed magnetic properties. The interface magnetic anisotropy is expected to decrease when the interface is not sharp. The present O-polar sample shows a rough Co/Pt interface but relatively strong PMA, suggesting that the existence of the Co-O bonding exerts an influence^39^. However, the Zn-polar sample shows a very sharp Zn/Co interface and clear in-plane interface anisotropy, indicating that the Zn/Co interface plays an important role in the magnetic anisotropy in this system. The negative *k*_surf._^ZnO/Co^ may be explained by the different electronic structure of the ML-Co on the Zn-polar surface^33^,^35^ and the anomalously larger atomic distance observed at the first ML-Co compared with the bulk (and/or its relaxation in the above layer). The *T*_C_ variation may be related to the difference in the degrees of intermixing between Co and Pt^40^ and between Co and O. The anisotropy difference may also affect the value of *T*_C_ in the present two-dimensional FM film^41^. The electrical charge-accumulation effect induced by the built-in electric field may also explain the observed differences. The electron density of Co is expected to increase (decrease) at the Zn-(O-)polar surface (Fig. 1b) because of the polarization effect^21^,^42^. Thus, from the perspective of charge accumulation, we note that the directions of the changes in magnetic anisotropy and *T*_C_ are consistent with our previous results obtained in the Pt/Co system under an applied external electric field^8^,^25^,^28^,^30^.

In summary, we found that the polarity of the surface of a NM polar oxide strongly affects the magnetic properties of a metallic thin film deposited on it. Merely depositing FM Co on either the front or back side (the Zn- or O-polar surface) of a ZnO substrate by sputtering under identical growth conditions completely changed the magnetic properties (the magnetic anisotropy and Curie temperature) of the Co film. The differences in crystallinity and/or electronic structure in the MLs of Co on the polar surfaces are expected to be relevant to the magnetic properties of the system. In particular, the Co on the Zn-polar surface shows high crystallinity and sharp interfaces. The structure considered here is expected to emerge as an ideal test platform for advanced spintronics research studies, *e.g*., the development of novel magnetic structures in which the Dzyaloshinskii–Moriya interaction is significant because of the lack of structural inversion symmetry^12^,^14^,^15^ or the Rashba spin-orbit-related phenomena^11^,^13^ originating from the built-in electric field at the Co atomic layer. Moreover, the results observed here constitute valuable information because the interface of a FM thin metal with an oxide has played and will probably continue to play a central role in the development of spintronic devices.

## Methods

### Sample preparation

The base pressure of the sputtering chamber used here (ES-280, EIKO Engineering Co. Ltd.) is the order of 10^−7^ Pa. RF-sputtering with Ar gas (0.7 Pa) was performed to deposit the Co and Pt cap layers on the ZnO substrates (Tokyo Denpa Co., Ltd.). The RF powers for the Co and Pt depositions were 60 W and 40 W, respectively. One ZnO substrate (1.5 × 1.5 cm^2^) was cut into two pieces. The metal layers were simultaneously deposited onto the front side of one piece and the back side of another (Zn- and O-polar samples) to ensure that the deposition conditions of each sample were the same. The nominal thickness was determined from the deposition rate of each layer.

### Hall effect measurement

**Figure 3:**
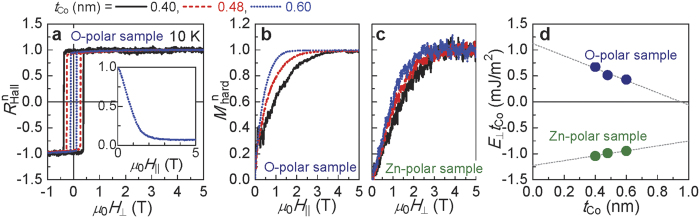
Magnetic anisotropy of the samples. (**a**) The normalized Hall resistance *R*_Hall_^n^ for the O-polar samples at 10 K obtained by sweeping the magnetic field perpendicular to the film (*μ*_0_*H*_⊥_). The inset shows the normalized Hall resistance under an in-plane magnetic field (*μ*_0_*H*_//_). Normalized hard-axis magnetization *M*_hard_^n^ curve for the (**b**). O-polar and (**c)**, Zn-polar samples at 10 K. (**d)**, The dependence of the PMA energy per unit area (*E*_⊥_*t*_Co_) on *t*_Co_ for both types of samples at 10 K.

The Hall measurement was performed using a Physical Property Measurement System (Quantum Design, inc.). To measure *R*_Hall_, a 30-*μ*m-wide Hall bar structure was fabricated via photolithography and Ar-ion milling. In the *R*_Hall_ curves shown in Fig. 3a, an odd-function component was extracted from the raw data, and then, the ordinary Hall resistance was removed from the extracted component.

## Additional Information

**How to cite this article**: Chiba, D. *et al*. Co thin films deposited directly on ZnO polar surfaces. *Sci. Rep*. **6**, 38005; doi: 10.1038/srep38005 (2016).

## Figures and Tables

**Figure 1:**
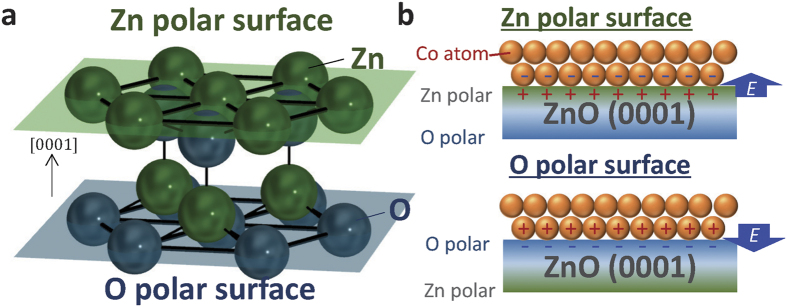
Zn- and O-polar surfaces of ZnO and the Co deposited directly on the ZnO. (**a**) Crystal structure of ZnO. (**b**) The same Co ultra-thin film deposited directly on the Zn- and O-polar surfaces of a ZnO(0001) substrate. The magnetic properties of the Co film are expected to vary depending on the polarity of the surface. One possible factor underlying this variation is the built-in electric field *E* in the interfacial Co atoms.

**Figure 2:**
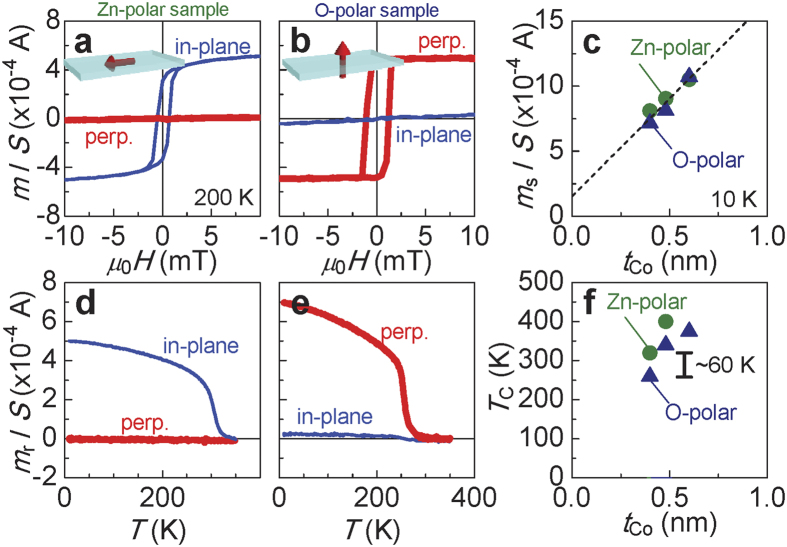
Magnetic properties of ultra-thin Co deposited on the ZnO polar surfaces. The magnetic moment per unit area (*m*/*S*) at 200 K for (**a)**, Zn- and (**b)**, O-polar samples with a Co thickness of *t*_Co_ = 0.4 nm. The red (blue) line indicates *m*/*S* obtained by sweeping an external magnetic field (*μ*_0_*H*) perpendicular to the plane (along the sample plane). (**c)** *t*_Co_ dependences of the saturation magnetic moment per unit area (*m*_s_/*S*) at 10 K. **(d,e)** The temperature dependence of the remanent magnetic moment per unit area (*m*_r_/*S*). (**f)** *t*_Co_ dependences of the Curie temperature *T*_C_.

**Figure 4:**
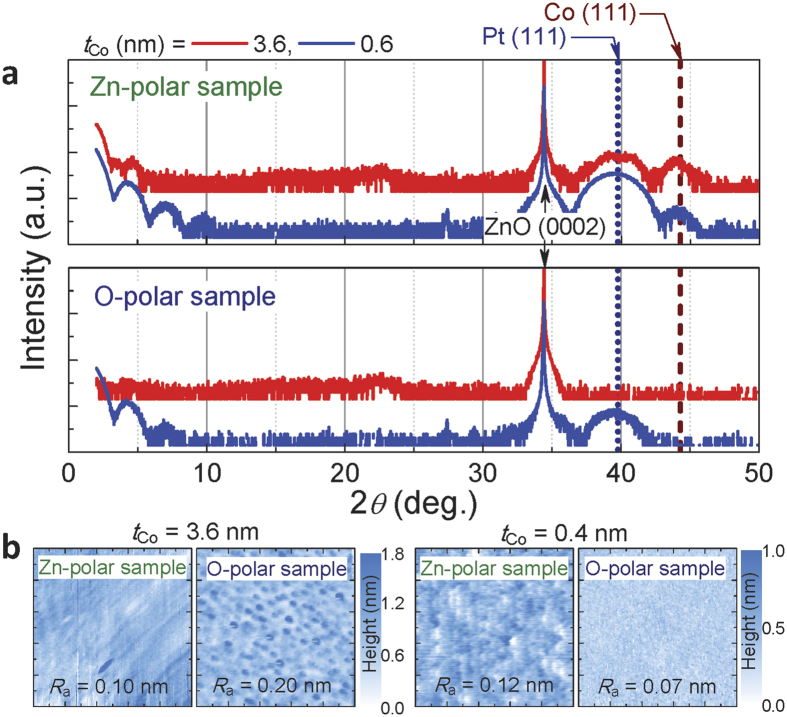
XRD profiles and AFM images. (**a**) XRD profiles for Zn- and O-polar samples with *t*_Co_ = 3.6 and 0.6 nm (red and blue lines, respectively). The vertical axis is in log scale. The dotted and dashed lines indicate the expected positions of the diffraction peaks from the fcc(111) planes for Pt and Co, respectively. (**b)** AFM images of Zn- and O-polar samples with *t*_Co_ = 3.6 and 0.4 nm. The scanned area was 1 × 1 *μ*m^2^.

**Figure 5:**
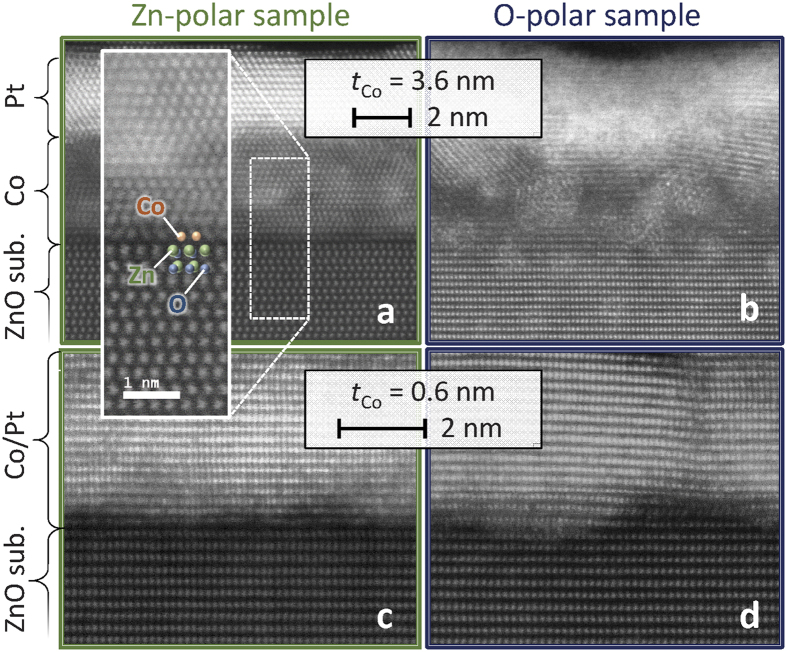
High-angle annular dark-field (HAADF) STEM images of the ZnO/Co interfaces. (**a**,**b**) The HAADF STEM images of Zn- and O-polar samples with *t*_Co_ = 3.6 and (**c**,**d**) 0.4 nm. The incident directions of the electron beam with respect to the ZnO substrate are [11
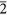
0] for (**a**) and [1
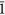
00] for (**b**–**d)**. The inset in **a** shows the magnified image of the area surrounded by the dotted line.

**Figure 6:**
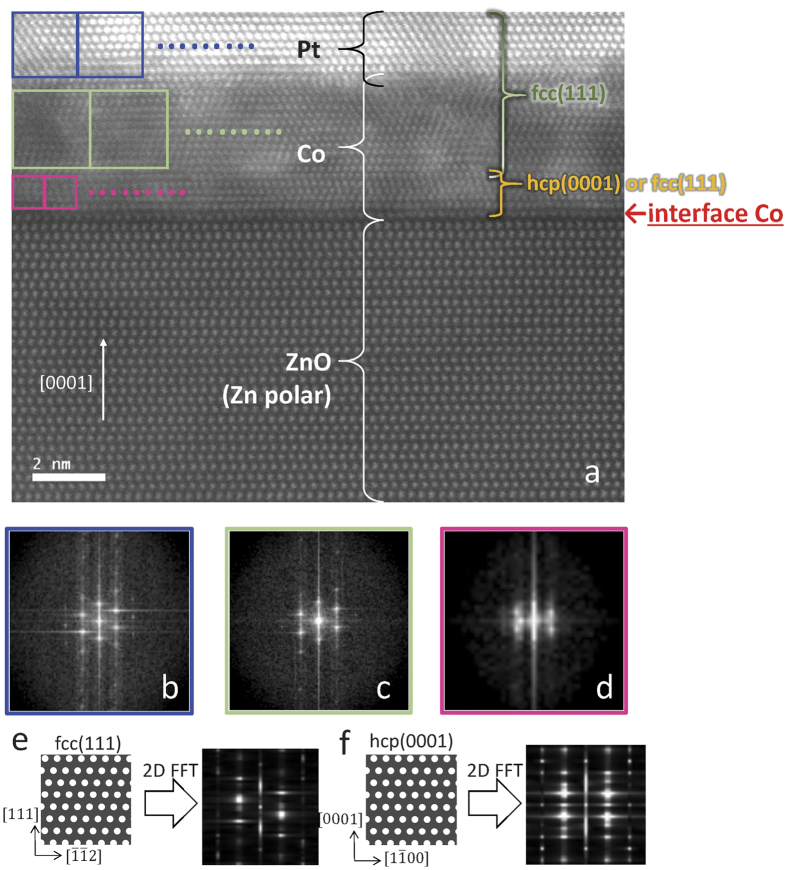
HAADF-STEM images of the Co and Pt layers deposited on the Zn-polar surface. (**a**) The HAADF-STEM image for a sample with *t*_Co_ = 3.6 nm. The incident direction of the electron beam with respect to the ZnO substrate is [11
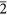
0]. The images of the 2D FFT for (**b**) the Pt layer, and (**c,d**) the Co layer. The square areas in the STEM image indicated by the blue (Pt), light-green (Co near the Co/Pt interface), and pink (Co near the ZnO/Co interface excluding the interface Co one ML) solid lines were used to perform the 2D FFT. Each 2D FFT image was averaged by the results obtained for the square areas arranged side by side, as shown in the STEM image. The simulation results of the 2D FFT for the artificially drawn (**e**) fcc(111) and (**f**) hcp(0001) structures.

**Figure 7:**
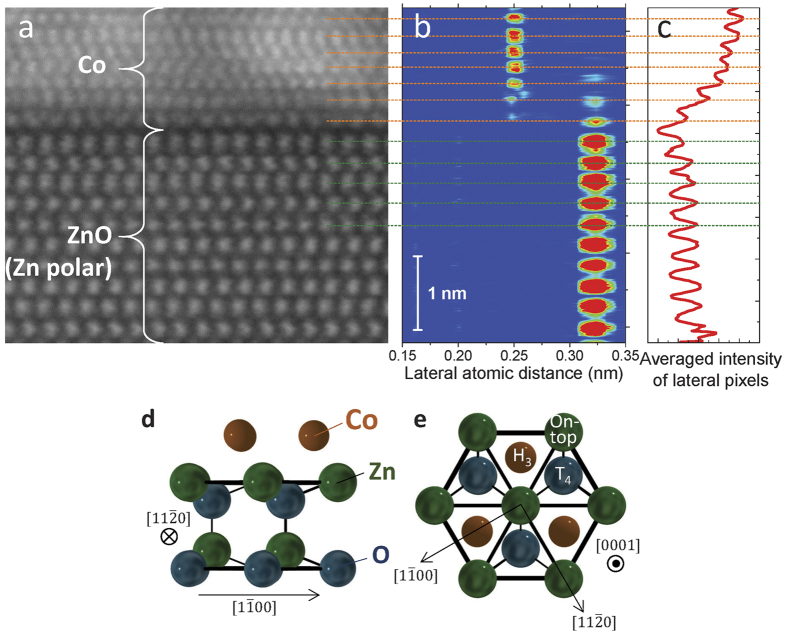
Determination of the atomic distance of the Co deposited on the Zn-polar surface. (**a**) The magnified HAADF-STEM image around the ZnO/Co interface for Zn-polar sample with *t*_Co_ = 3.6 nm. (**b**) The FFT intensity map for the corresponding lateral pixels in the STEM image. The colour indicates the intensity (red: high, blue: low). The peak position corresponds to the LAD. The interfacial ML of Co shows exactly the same LAD as the surface Zn of the ZnO substrate. (**c**) The averaged intensity of the lateral pixels of the STEM image. The intensity just below the interface Co atoms is the weakest in the image. The (**d**) side view and (**e)** top view of the schematic image depicting the expected positions of the Co atoms on the Zn-polar surface.

**Figure 8:**
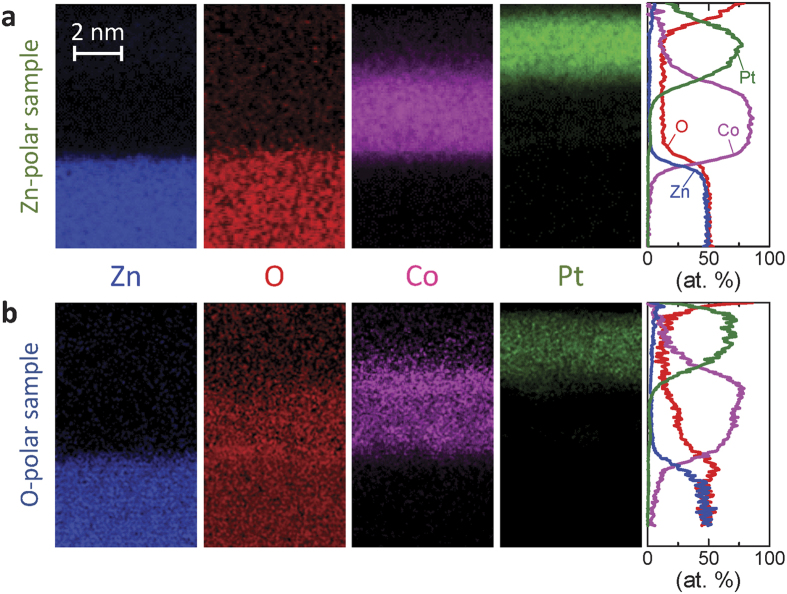
The elemental mappings obtained by STEM-EDS. The panels show the EDS mappings of Zn, O, Co, and Pt from left to right in (**a**) Zn- and (**b**). O-polar samples with *t*_Co_ = 3.6 nm. The graphs on the right show the averaged line profiles of the maps for each element.
